# Accidents

**Published:** 1882-01

**Authors:** 


### ACCIDENTS

MEAN, literally, falling to or coming upon, by agencies beyond our control. The ancients regarded them as coming from heaven, and the idea has descended to modern time, and is expressed by the word providential.

Providentially hindered is a frequent phrase, but the accident which brings harm to a man's body will be found on investigation to be the result of ignorance, carelessness or design on the part of the injured, or some other person. It is called a railroad accident if a switch has been designedly misplaced; it is regarded as an accident if a person's clothing takes fire from the bursting of a kerosene lamp, when in reality it resulted in the ignorance or carelessness of the person handling it, in great part, but not altogether; the man who supplied the oil is in part a criminal for supplying a burning fluid which is dangerous, and which if honestly prepared would not have taken fire. It was his business to have ascertained by his own testing that the oil was safe, which he could have done without expense or trouble in five minutes.

In most accidents or casualties there are several things to be done at once, and in many cases these first things decide the issue of life and death.

*First.* Nine times in ten brandy or wine or other forms of stimulant are almost instinctively offered. If drink is asked for give cold water ; if not asked for, wait.

*Second.* If anything is likely to obstruct the breathing, as tight clothing or mud or other thing about the face and nose, remove it and place the person in a reclining position ; if the brain or head is not injured, on the back : and beyond two, or at most three, persons, keep everyone away at least ten feet distant, and, a great deal better, out of the room altogether, for the very sight of many persons present tends to excite and alarm and discompose by the expression of their countenances alone. In very many cases the injured person is more calm and self-possessed than anyone present, and, for an additional reason, a crowd should be kept at a distance to allow abundant pure air to get to the sufferer.

*Third.* Before moving the person notice if any limb is broken, or if there is much blood flowing ; if flowing in spurts or jets, an artery has been severed, and there is no time to be lost in moving, for the person may bleed to death in a few moments. If there is not much blood discharged, and it comes out slowly, removal can take place ; but then it is generally better to promote the bleeding by the use of a sponge and warm water, for in most accidents moderate bleeding unloads the system and tends to prevent inflammation and erysipelas from setting in.

*Fourth.* Do all that is possible to compose the patient, to give rest to the body, to each limb, and by all means to keep the clothing dry and the body and extremities warm ; this is always of consequence, always of vital importance. In addition keep out of the sufferer's sight everything which might excite or discompose or discourage or alarm ; remove everything bloody, everything soiled or torn.

*Fifth.* The persons around the patient should exhibit a quiet, confident, composed, assured air, so as to inspire the same in the mind of the sufferer.

*Sixth.* Avoid all sudden motions or starts or hurry ; avoid noisy talking, especially all whispering, which is a pest to any chamber ; do not allow the patient to observe you steadily gazing at him, and ask him as few questions as possible ; try and find out what is wanting without inquiry.

*Seventh.* In all cases of injury by accidents send for the nearest physician without a moment's delay ; if one cannot be had, send for a nurse ; if none at hand, send for the woman nearest who has had most experience in nursing the sick.

*Eighth.* Until a physician or other help arrives, have two basins of cold water and half a dozen soft rags, and wherever there is a wound or bruise or swelling apply the rags dipped in the water ; the object being to keep down fever and inflammation. Bear in mind, to cool and to cleanse, warm water is sometimes better than cold ; it does not give such a shock, to begin with ; but, whether warm or cold, be careful to prevent the water from dribbling about on the clothing or bed, both of which should be kept perfectly dry.

If the person is insensible, apply camphor to the nostrils, and cloths dipped in hot water to the pit of the stomach ; rub the limbs vigorously with the hands or hot, dry towels ; bathe the temples and forehead in water and vinegar, half and half.—*Dr. Hall's Health at Home.*

